# Resistance mechanisms and genetic relatedness among carbapenem-resistant *Pseudomonas aeruginosa* isolates from three major hospitals in Hanoi, Vietnam (2011–15)

**DOI:** 10.1093/jacamr/dlab103

**Published:** 2021-07-27

**Authors:** Hai Anh Tran, Thi Ngoc Bich Vu, Son Tung Trinh, Dieu Linh Tran, Ha My Pham, Thi Hong Hanh Ngo, Minh Thao Nguyen, Nhu Duong Tran, Duy Thai Pham, Duc Anh Dang, Keigo Shibayama, Masato Suzuki, Lay-Myint Yoshida, Hong Son Trinh, Viet Thanh Le, Phuong Thom Vu, Thi Vu Nga Luu, Anne-Laure Bañuls, Khanh Linh Trinh, Van Anh Tran, Huy Hoang Tran, H Rogier van Doorn

**Affiliations:** 1 Hanoi Medical University, Hanoi, Vietnam; 2 Oxford University Clinical Research Unit, Hanoi, Vietnam; 3 National Institute of Hygiene and Epidemiology, Hanoi, Vietnam; 4 National Institute of Infectious Diseases, Tokyo, Japan; 5 Institute of Tropical Medicine, Nagasaki University, Nagasaki, Japan; 6 Viet-Duc Hospital, Hanoi, Vietnam; 7 Quadram Institute Bioscience, Norwich Research Park, Norwich, UK; 8 Saint Paul Hospital, Hanoi, Vietnam; 9 Thanh Nhan Hospital, Hanoi, Vietnam; 10 MIVEGEC Univ Montpellier-IRD-CNRS, Centre IRD, Montpellier, France; 11 LMI DRISA, Hanoi, Vietnam; 12 High School for Gifted Students, Hanoi University of Science; 13 Centre for Tropical Medicine and Global Health, Nuffield Department of Clinical Medicine, University of Oxford, Oxford, UK

## Abstract

**Background:**

MDR bacteria including carbapenem-resistant *Pseudomonas aeruginosa* are recognized as an important cause of hospital-acquired infections worldwide. This investigation seeks to determine the molecular characterization and antibiotic resistance genes associated with carbapenem-resistant *P. aeruginosa*.

**Methods:**

We conducted WGS and phylogenetic analysis of 72 carbapenem-resistant *P. aeruginosa* isolated from hospital-acquired infection patients from August 2011 to March 2015 in three major hospitals in Hanoi, Vietnam.

**Results:**

We identified three variants of IMP gene, among which *bla*_IMP-15_ was the most frequent (*n *=* *34) in comparison to *bla*_IMP-26_ (*n *=* *2) and *bla*_IMP-51_ (*n *=* *12). We observed two isolates with imipenem MIC >128 mg/L that co-harboured *bla*_IMP-15_ and *bla*_DIM-1_ genes and seven isolates (imipenem MIC > 128 mg/L) with a *bla*_KPC-1_ gene from the same hospital. MLST data shows that these 72 isolates belong to 18 STs and phylogenetic tree analysis has divided these isolates into nine groups.

**Conclusions:**

Our results provide evidence that not only *bla*_IMP-26_ but other IMP variants such as *bla*_IMP-15_ and *bla*_IMP-51_ genes and several STs (ST235, ST244, ST277, ST310, ST773 and ST3151) have been disseminating in healthcare settings in Vietnam. In addition, we report the emergence of two isolates belonging to ST1240 and ST3340 that harboured two important carbapenemase genes (*bla*_IMP-15_ and *bla*_DIM-1_) and seven isolates belonging to ST3151 of *P. aeruginosa* that carried the *bla*_KPC-1_ gene in Vietnam, which could potentially cause serious restricted availability of treatment options in healthcare settings.

## Introduction

Antibiotic resistance has taken centre stage as a global health issue that demands public attention. Concerns have been raised due to the rapid emergence and spread of carbapenem-resistant Gram-negative bacteria resistant to the antibiotic group used as a ‘last resort’ in hospital treatments. In addition, bacteria have been found to be resistant to colistin, which is recommended to be used as salvage treatment for infections caused by carbapenem-resistant bacteria.^1–3^ With the emergence of resistance to these drugs, there might be no effective antibiotic treatment for these bacteria in the next 5–10 years.

MDR *Pseudomonas aeruginosa* is recognized as an important cause of hospital-acquired infections and is listed among the WHO priority pathogens for research and development of new antibiotics.^4^^,^^5^ This bacterium is highly adaptable to environmental fluctuations, including low-level antibiotic exposure, and many antibiotic resistance mechanisms such as reduced membrane permeability, drug efflux pumps and enzymatic inactivation have been found. The spread of antibiotic resistance genes through mobile genetic factors greatly contributes to the formation of antibiotic-resistant *P. aeruginosa*,^6^^,^^7^ which is well known to have simultaneous multiple resistance mechanisms thus limiting treatment choices.^8–10^ Epidemiological classification of *P. aeruginosa* using PFGE has been used as the gold standard for molecular epidemiology to characterize and identify the risk of transmission and spread of *P. aeruginosa* outbreaks in hospitals.^11^ However, this technique has limited discriminatory capacity, high cost and complex workflow, and does not provide detailed information on the evolutionary background of *P. aeruginosa*. Currently, next-generation sequencing usage is becoming broader as it provides data not only on the genetic relatedness at a higher resolution but also on resistance-associated genes and their relatedness and thus more insights into antimicrobial-resistant bacteria. With this technique, the relatedness and transmission of hospital isolates can be assessed and used to guide infection control interventions locally. Moreover, sequence and evolutionary analyses contribute to enhance the global picture, the temporal and spatial evolution of antibiotic resistance genes and associated bacteria.^12^^,^^13^

Southeast Asia is considered to be a ‘hot spot’ of antibiotic-resistant bacteria and *P. aeruginosa* has also been identified as a common cause of hospital-acquired infections in Vietnam.^14^^,^^15^ According to statistics from the Center for Disease Dynamics, Economics & Policy (CDDEP) in 2016, 36% of *P. aeruginosa* isolates in Vietnam were resistant to carbapenems, ranking second only after India.^14^ Nevertheless, little is known regarding the *P. aeruginosa* genotypes and antibiotic resistance gene types circulating in Vietnam. This information is important since the STs of *P. aeruginosa* associated with antibiotic resistance genes differ markedly among communities, hospitals and countries.^16–19^ A study in one hospital in Hanoi, Vietnam reported a carbapenemase-ST235 *P. aeruginosa* carrying *bla*_IMP-15_*, bla*_IMP-26_, and *bla*_IMP-51_ genes.^16^ The *P. aeruginosa* ST235 isolates were identified as playing an important role in relation to hospital-acquired infections. To further our knowledge and provide a better understanding of the circulation of these highly drug-resistant pathogens, we conducted surveillance of carbapenem-resistant *P. aeruginosa* in three major hospitals in Hanoi between August 2011 and March 2015. Here we present phenotypic and genotypic data on IMP-positive isolates from this collection from Vietnam, a middle-income country with a high and increasing burden of antimicrobial resistance (AMR) and hospital-acquired infection and compare these with local, regional and global data to add the current knowledge of carbapenem-resistant *P. aeruginosa*.

## Methods

### Hospital settings and isolates

Isolates were collected from three hospitals (Saint Paul, Thanh Nhan and Viet Duc) with high capacity, located in the centre of Hanoi, the capital city of Vietnam. Saint Paul and Thanh Nhan hospitals are reference healthcare settings with over 600 bed capacity each and include many specialities such as surgery, paediatrics and ICU. Viet Duc Hospital is the largest surgical centre in Vietnam with over 1500 beds and performs different types of surgery, such as abdominal, gastroenterology and hepatobiliary, paediatric and urology. Demographic and basic clinical information of patients whose specimens were carbapenem-resistant *P. aeruginosa* positive were collected from clinical notes and included age, gender, date of admission, clinical diagnosis, the origin of the collected sample, isolated bacterial strains and date of sample collection. Treatment and clinical outcome data were not available for this study.

*P. aeruginosa* isolated from clinical specimens were tested for antibiotic susceptibility using disc diffusion testing according to international guidelines.^20^ The microbiology consultants of the hospitals were requested to collect and send all bacterial isolates resistant at least to one of the antibiotics in the carbapenem group to the National Institute of Hygiene and Epidemiology (NIHE) to detect carbapenem resistance genes by PCR. From the collection of carbapenem-resistant *P. aeruginosa* isolates (*n *=* *416) sent between August 2011 and March 2015, we screened by PCR for the presence of five common carbapenemase genes (*bla*_IMP_*, bla*_VIM_*, bla*_SIM_*, bla*_SPM_ and *bla*_NDM-1_).^2^^,^^21^ The PCR results were then reported to the hospitals. Based on the PCR screening results, we conducted WGS analysis of some carbapenem-resistant isolates for further characterization. Seventy-two non-repetitive isolates were selected for this study, including all the isolates positive for *bla*_IMP_ genes (*n *=* *48; 18 isolates from Saint Paul, 11 from Thanh Nhan and 19 from Viet Duc), and 24 isolates negative for *bla*_IMP_, *bla*_VIM_*, bla*_SIM_*, bla*_SPM_ and *bla*_NDM-1_ genes by PCR (isolates were selected from same wards and years as PCR positive isolates: 12 isolates from Saint Paul, 8 from Thanh Nhan and 4 from Viet Duc).

### Bacterial identification and susceptibility testing

The species was confirmed by the MALDI Biotyper system (Bruker Daltonik GmbH, Germany). MIC testing of seven antibiotics commonly used in the treatment of *P. aeruginosa* infections in Vietnam was performed by agar dilution for imipenem, ceftazidime, ciprofloxacin, gentamicin, amikacin and aztreonam (Sigma–Aldrich) and by broth microdilution for colistin. The susceptibility testing procedure was conducted according to CLSI guidelines 2018 using *P. aeruginosa* ATCC27853 as a quality control strain.^20^

### WGS of P. aeruginosa

To prepare WGS libraries, genomic DNA of 72 *P. aeruginosa* isolates was extracted using QIAamp DNA Mini Kit (Qiagen, Hilden, Germany) according to the manufacturer’s protocol. Libraries of *P. aeruginosa* strains were prepared using the Nextera XT DNA Library Prep Kit (Illumina, San Diego, CA, USA). Multiplexed paired-end sequencing was performed using the MiSeq Reagent V3 Kit (2 × 300 cycles) on an Illumina MiSeq instrument.

### Bioinformatics analysis

Whole-genome sequences were analysed using an in-house bioinformatics pipeline, which runs on a Conda environment under Linux. Briefly, we used FastQC Version 0.11.8 for quality control of raw reads. Reads were trimmed of the adaptor sequences and were subsequently *de novo* assembled into contigs using SPAdes (3.9.0) with a pre-defined Kmers set. AMR genes were identified from the assembled contigs using the ABRicate program to query the Resfinder database V2.1.^22^

MLST was conducted from the Shovill-output contigs, screening seven housekeeping genes against the PubMLST database. Alleles were submitted to the PubMLST database to get the ST. A phylogenetic tree based on the core genome SNPs was constructed from WGS data of the 72 *P. aeruginosa* isolates using Parsnp 1.2 and IQ-TREE 1.5.^22^^,^^23^

### Ethics approval and consent to participate

Ethics approval was obtained from the Ethics Committee of the Vietnamese National Institute of Hygiene and Epidemiology for the main project ‘Assessing the impact and burden of antimicrobial resistance in Vietnam, genomic characterization and risk factors related to antimicrobial resistance of common bacteria in Vietnam’. Individual informed consent was waived because of the retrospective nature of this work and because no patient identifying information was collected (IRB code: IRB-VN01057-38/2016).

### Availability of data and materials

All data generated or analysed during this study are included in this article (and its supplementary data files).

## Results

### Characterization of P. aeruginosa

Four hundred and sixteen carbapenem-resistant *P. aeruginosa* isolates were collected from three hospitals between August 2011 and March 2015. A total of 48 IMP-gene PCR positive and 24 PCR negative isolates from the same wards and years were further characterized using WGS.

**Figure 1.**
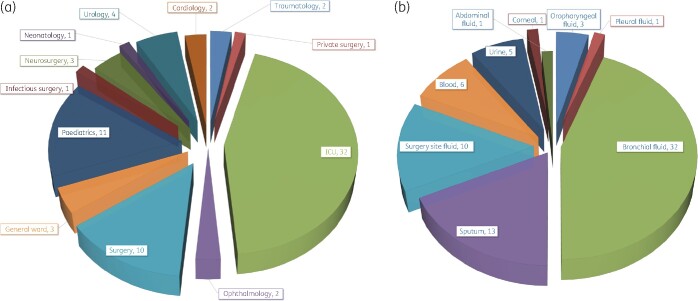
Distribution by department (a) and source and type of *P. aeruginosa* isolates (b) in three hospitals of Hanoi (*n *=* *72).

The median age of these 72 patients with *P. aeruginosa* infections was 35 years (range: 1 to 85 years) and the ratio of male to female was 2.6. Forty-five isolates (45/72; 62.5%) were isolated from pneumonia patients including 29 ventilator-associated pneumonia (VAP) patients. These isolates were detected from 11 different departments, with a majority from the ICU (44.4%, *n *=* *32), paediatrics (15.2%, *n *=* *11) and surgery (13.9%, *n *=* *10) (Figure 1). The *P. aeruginosa* isolates were cultured from eight types of sample, including bronchial fluid (44.4%, *n *=* *32), sputum (18%, *n *=* *13) and surgical site fluid (13.9%, *n *=* *10) (Figure 1).

### Profile of antibiotic resistance genes

**Figure 2.**
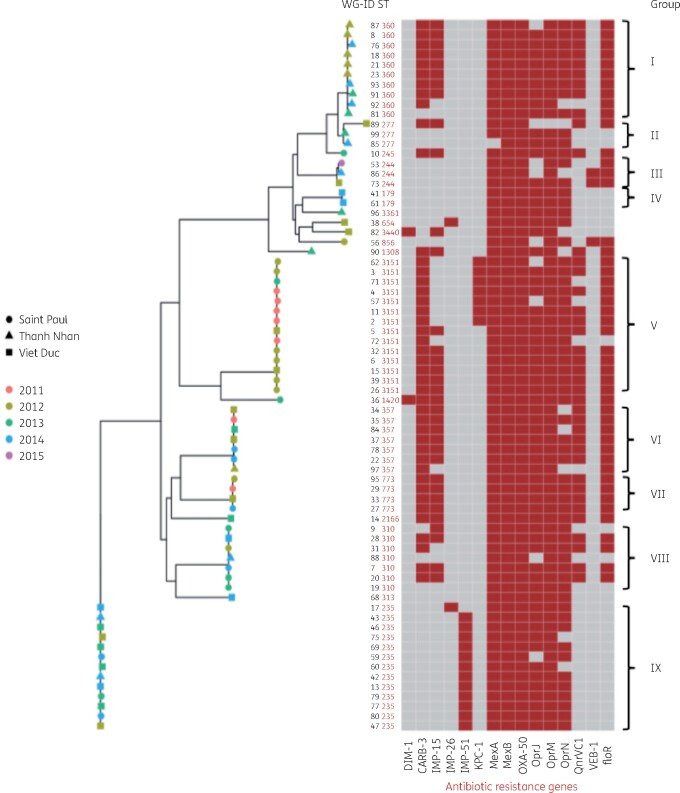
Core genome phylogenetic tree of the 72 *P. aeruginosa* isolates of the three hospitals associated with STs and antibiotic resistance genes. The shapes represent the different hospitals, colours indicate the collection year of the isolate and red squares indicate the presence of AMR genes in isolates.

*P. aeruginosa* clinical isolates displayed different combinations of drug resistance genes such as efflux pump and outer membrane (*mexAB-oprM, mexEF-oprN, oprJ, opmB, opmH*) genes (Figure 2) leading to multidrug resistance. All the 72 *P. aeruginosa* isolates under study carried *bla*_OXA-50_, *fosA* (fosfomycin resistance) and different variants of *Pseudomonas*-derived cephalosporinase (PDC)-β-lactamase class C genes, predominantly *bla*_PDC-2_ (18/72, 25%) and *bla*_PDC-7_ (18/72, 25%), followed by *bla*_PDC-3_ (17/72, 24%), *bla*_PDC-5_ (15/72, 21%), *bla*_PDC-1_ (2/72, 3%) and *bla*_PDC-8_ (2/72, 3%). Other genes encoding antibiotic resistance were found in this study including genes for CARB-3-β-lactamase (*CARB-3,* 43/72, 60%), acquired fluoroquinolone resistance (*qnrVC1*, 37/72, 51%) and Vietnamese extended-spectrum β-lactamase (*bla*_VEB-1_, 3/72, 4%). Among the 48 IMP-gene PCR positive isolates, *bla*_IMP-15_ was the most frequently detected (34/48, 71%), followed by *bla*_IMP-51_ (12/48, 25%) and *bla*_IMP-26_ (2/48, 4%) (Table 1, Table S1 and Figure S1, available as Supplementary data at *JAC-AMR* Online).

**Table 1.** Distribution of antibiotic resistance genes of *P. aeruginosa* (*n *=* *72)

Carbapenem genes	Important AMR genes						
	DIM-1	OXA-50	PDC (PDC-β-lactamase class C)	CARB-3	VEB-1	QnrVC1	FosA
IMP (*n *=* *48)							
*bla*_IMP-15_ (*n *=* *34)	2/34	34/34	*bla*_PDC-2_ (*n *=* *4); *bla*_PDC-3_ (*n *=* *10); *bla*_PDC-5_ (*n *=* *11); *bla*_PDC-7_ (*n *=* *9)	32/34	—	31/34	30/34
*bla*_IMP-26_ (*n *=* *2)	—	2/2	*bla*_PDC-2_; *bla*_PDC-3_	—	—	1/2	2/2
*bla*_IMP-51_ (*n *=* *12)	—	12/12	*bla*_PDC-2_ (*n *=* *12)	—	—	—	12/12
*bla*_KPC-1_ (*n *=* *7)	—	7/7	*bla*_PDC-7_ (*n *=* *7)	7/7	—	5/7	7/7
IMP, KPC-negative isolates (*n *=* *17)	—	17/17	*bla*_PDC-1_ (*n *=* *2); *bla*_PDC-2_ (*n *=* *1); *bla*_PDC-3_ (*n *=* *6); *bla*_PDC-5_ (*n *=* *4); *bla*_PDC-7_ (*n *=* *2); *bla*_PDC-8_ (*n *=* *2)	4/17	3/17	—	17/17
Total, *n*/*N* (%)	2/72 (2.77)	72/72 (100)	72/72 (100)	43/72 (59.72)	3/72 (4.16)	37/72 (51.38)	72/72 (100)

Notably, we detected the presence of the *bla*_DIM-1_ gene (Dutch imipenemase 1) encoding carbapenemase, to our knowledge for the first time in Vietnam, in two *P. aeruginosa* isolates. The first *bla*_DIM-1_-carrying *P. aeruginosa* isolate was from the urine of a posterior urethral stenosis patient in the private surgery department of Viet Duc hospital in mid-July 2012. The second *bla*_DIM-1_-harbouring isolate was from an ICU pneumonia patient in Saint Paul hospital in early 2013. Noticeably, both isolates with *bla*_DIM-1_ genes also carried the *bla*_IMP-15_ gene. Patients with *bla*_DIM-1_-positive isolates were treated in two different hospitals, and these isolates belonged to ST1420 and ST3440 (Figure 2). We detected seven *bla*_KPC-1_-positive *P. aeruginosa* isolates belonging to ST3151, all in Saint Paul hospital between 2011 and 2013 in the paediatrics (*n *=* *3), ICU (*n *=* *3) and ophthalmology (*n *=* *1) departments. The first *bla*_KPC-1_-positive isolate was in October 2011 from the bronchial fluid of a VAP patient. The second and third isolates were from the blood of a septic patient and the corneal sample of a conjunctivitis patient in November 2011 and the fourth was from a pneumonia patient in December 2011. Two *bla*_KPC-1_-positive strains were isolated in May 2012 from the bronchial fluid of pneumonia patients and the last case in January 2013.

### Antimicrobial susceptibility of P. aeruginosa

The results of the imipenem MIC tests showed that the isolates without *bla*_IMP-15_*, bla*_IMP-26_*, bla*_IMP-51_*, bla*_DIM-1_ or *bla*_KPC-1_ genes had the lowest MICs (8–16 mg/L). Isolates carrying only the *bla*_IMP-51_ gene had an MIC of 16–32 mg/L, and isolates carrying only the *bla*_IMP-15_ gene had MICs of 32–64 mg/L. Noticeably, isolates with both *bla*_IMP-15_ and *bla*_DIM-1_ genes were extremely resistant to imipenem, with MICs >128 mg/L. Similarly, isolates carrying the *bla*_KPC-1_ gene had MICs >128 mg/L. We also observed that isolates co-carrying *bla*_IMP-15_ and *bla*_DIM-1_ or carrying only one gene among *bla*_IMP-26_*, bla*_IMP-51_ and *bla*_KPC-1_ were resistant to five other antibiotics tested (ciprofloxacin, ceftazidime, gentamicin, amikacin and aztreonam) (Table 2). However, most of the isolates carrying the *bla*_IMP-15_ gene (18/32) were still susceptible to aztreonam, and isolates carrying both *bla*_IMP-26_ and *bla*_DIM-1_ genes were amikacin susceptible. Among 17 isolates not harbouring acquired carbapenemase genes (*bla*_IMP_*, bla*_DIM-1_ and *bla*_KPC-1_), 16 isolates remained susceptible to ciprofloxacin (0.125–1 mg/L); amikacin (2–4 mg/L) and gentamicin (1–2 mg/L), 12 to aztreonam (8 mg/L) and 10 to ceftazidime (4 mg/L). Finally, we observed 8 of 72 isolates resistant to colistin with MICs ranging from 4 to 16 mg/L. (Table 2, Table S1).

**Table 2.** Antimicrobial susceptibility by MIC of *P. aeruginosa* (*n *=* *72)

*P. aeruginosa* strains (*n *=* *72)	Susceptibility (MIC, mg/L)						
	IPM	CIP	CAZ	AMK	GEN	ATM	CST
*bla*_IMP-15_ (*n = *32)	R (32–64)	R (4–16)	R (64–256)	S (8), *n *=* *1; R (>256), *n *=* *31	R (>128)	S (2–8), *n *=* *18; I (16), *n *=* *13; R (32), *n *=* *1	S (0.25–2), *n *=* *28; R (4–16), *n *=* *4;
*bla*_IMP-15_ *+bla*_DIM-1_ (*n = *2)	R (>128)	R (16)	R (>256)	S (16)	R (>128)	R (128)	S (0.25–2), *n *=* *2
*bla*_IMP-26_ (*n = *2)	R (>128)	R (32)	R (>256)	R (>256)	R (>128)	R (32)	S (0.5), *n *=* *1; R (4), *n *=* *1
*bla*_IMP-51_ (*n = *12)	R (16–32)	R (8–16)	R (256)	S (8–16), *n *=* *6; R (64)	R (32–64)	I (1), *n *=* *2; R (32–128), *n *=* *5	S (0.25–2), *n *=* *11; R (4), *n *=* *1
KPC-1 (*n = *7)	R (>128)	R (8)	R (32)	R (>256)	R (>128)	R (>128)	S (0.125–2), *n *=* *6; R (4), *n *=* *1
IMP, DIM KPC- negative (*n = *17)	R (8–16), *n *=* *17	S (0.125–1), *n *=* *16; R (64), *n *=* *1	S (4), *n *=* *10; R (32–128), *n *=* *7	S (2–4), *n *=* *16; R (>256), *n *=* *1	S (1–2), *n *=* *16; R (32); *n *=* *1	S (8), *n *=* *12; I (16), *n *=* *3; R (>128), *n *=* *2	S (0.25–2), *n *=* *15; R (4), *n *=* *1

IPM, imipenem; CIP, ciprofloxacin; CAZ, ceftazidime; AMK, amikacin; GEN, gentamicin; ATM, aztreonam; CST, colistin; S, susceptible; I, intermediate; R, resistant.

### Genotypic relationship of P. aeruginosa isolates

**Figure 3.**
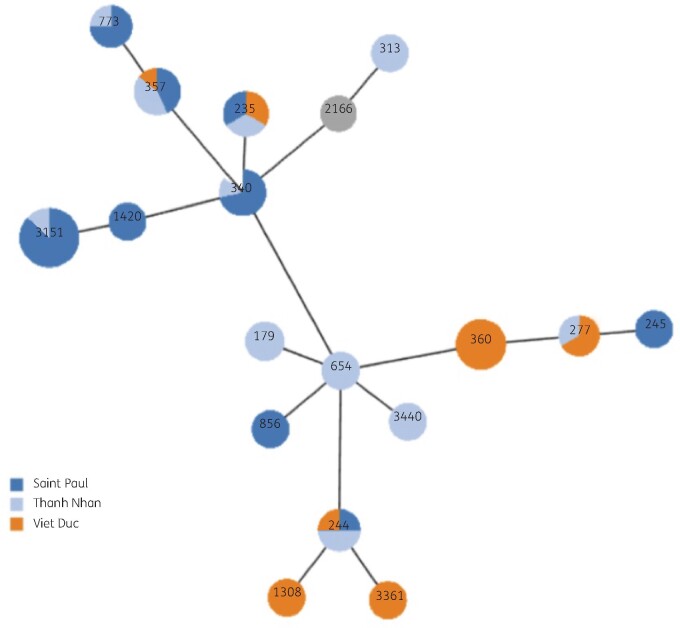
Spanning tree of *P. aeruginosa* using MLST data reported in this study*.* Each circle and number represents one ST; the colour indicates the hospital.

The phylogenetic tree placed the 72 *P. aeruginosa* isolates in nine genotype groups (Figure 3). Each group had different characteristics of antibiotic resistance genes and STs. Interestingly, group I included 10 potentially clonal Thanh Nhan hospital isolates from 2012–14 belonging to ST360, with 8/10 isolates harbouring *bla*_IMP-15_, and most of the isolates carried many different resistance-encoding genes: *bla*_OXA-50_*, CARB-3, bla*_PDC_ (PDC-β-lactamase class C) and a gene encoding quinolone resistance (*qnrVC1*). Group V isolates belonged to ST3151 and harboured one or a combination of two carbapenemase genes including *bla*_IMP-15_*, bla*_KPC-1_*, bla*_OXA-50_*, qnrVC1, CARB-3* and *bla_PDC_* genes. Group IX was ST235, these strains carried *bla*_IMP-51_ and *bla*_OXA-50_ genes and were found in all three hospitals between 2012 and 2014 (Table S1, Figure 2).

MLST of *P. aeruginosa* isolates showed that the 72 isolates were classified into 18 STs*,* among which ST3151 was most often detected (*n *=* *14) followed by ST235 (*n *=* *13), ST360 (*n *=* *10), ST310 (*n *=* *7) and ST357 (*n *=* *7). The remaining 13 singletons included ST179, ST244, ST245, ST277, ST313, ST654, ST773, ST856, ST1308, ST1420, ST2166, ST3440 and ST3361. Interestingly, all ST360 isolates (10/10) were detected from patients in Thanh Nhan Hospital. ST3151 and ST310 were found significantly more often in Saint Paul Hospital compared with Viet Duc Hospital (13/15 versus 2/15 and 5/7 versus 1/7, respectively) (Figures 2 and 3).

## Discussion

In this study, we collected phenotypically carbapenem-resistant isolates of *P. aeruginosa* from three large hospitals in Hanoi, Vietnam between 2011 and 2015. Out of these 416 isolates, we further characterized 48 isolates PCR positive for *bla*_IMP_ genes and 24 PCR negative for *bla*_IMP_ and *bla*_VIM_*, bla*_SIM_*, bla*_SPM_ and *bla*_NDM-1_ genes. The majority of the 72 carbapenem-resistant *P. aeruginosa* infection cases in this study were from nosocomial infections, mostly healthcare-associated pneumonia (*n *=* *45 including 29 VAP) and 10 surgical site infections. Our observations suggest that potential dissemination of carbapenem-resistant *P. aeruginosa* may occur at different departments in these hospitals, either by transferring between patients or through healthcare workers.

Antimicrobial resistance among key pathogens in Vietnam is high and increasing, especially among *Escherichia coli* and ESKAPE organisms (*Enterococcus faecium*, *Staphylococcus aureus*, *Klebsiella pneumoniae*, *Acinetobacter baumannii*, *P. aeruginosa* and *Enterobacter* spp.), especially those associated with nosocomial infection. Data from a nationwide hospital surveillance network show high and increasing resistance among *P. aeruginosa* with proportions reported to be resistant against carbapenems of 32% in 2012–13 and 44% in 2016–17.^24^ The results of our study should be interpreted against that background of high and increasing levels of resistance among pathogens associated with nosocomial infection, including *P. aeruginosa*.

Our study found that *P. aeruginosa* carried different combinations of antibiotic resistance genes leading to a broad spectrum of resistance to various antibiotics. The 48 *P. aeruginosa* isolates with *bla*_IMP_ genes revealed three different IMP variants (*bla*_IMP-15_, *bla*_IMP-51_ and *bla*_IMP-26_) that have been reported from other countries including Mexico, Korea and Singapore.^17^^,^^18^^,^^25^ The dominance of these three genes was also similar to a study conducted simultaneously in the largest hospital in Hanoi, Vietnam (2013–14).^16^ Our results showed that *bla*_IMP-15_ and *bla*_IMP-51_ were most frequently detected and support the evidence that these variants of *bla*_IMP_ genes have been disseminated in Vietnamese healthcare settings.

The *bla*_DIM-1_ gene was detected, to our knowledge for the first time in Vietnam, in two *P. aeruginosa* isolates from Saint Paul and Viet Duc hospitals and belonged to different STs: ST1420 and ST3440. Previous studies have shown that the *bla*_DIM-1_ gene encodes a group of B MBL enzymes capable of lysis of carbapenem antibiotics that was discovered in the integron class 1 genetic element (*intl1*) of *Pseudomonas stutzeri* in the Netherlands in 2007, in *P. aeruginosa* in India (5%, 2010) and in Sierra Leone (46.7%, 2013).^26^^,^^27^

We also found 7 out of 72 *P. aeruginosa* isolates carrying *bla*_KPC-1_ belonging to ST3151. Genes for KPC have previously been reported, albeit rarely, in *P. aeruginosa* in China and the USA.^28^^,^^29^ Additionally, we found a plasmid carrying the *bla*_KPC_ gene in Enterobacteriaceae clinical isolates from Saint Paul Hospital in 2010 and from other hospitals in Vietnam, which are currently being characterized, suggesting that the KPC-1-encoding gene of the *P. aeruginosa* isolates in the study might be acquired from *K. pneumoniae* and *E. coli* through conjugation. The combination of IMP genes with either DIM or KPC genes led to very high (>128 mg/L) carbapenem MICs.

The imipenem MIC values for the *bla*_IMP-15_-, *bla*_IMP-26_-, *bla*_IMP-51_-, *bla*_DIM-1_- and *bla*_KPC-1_-positive *P. aeruginosa* isolates ranged from 16 to >128 mg/L. These MIC values have been documented for carbapenemase-producing *P. aeruginosa* in other studies.^26–29^ The isolates without *bla*_IMP-15_, *bla*_IMP-26_, *bla*_IMP-51_, *bla*_DIM-1_ and *bla*_KPC-1_ had MICs of 8–16 mg/L. Previous studies showed that carbapenem-resistant *P. aeruginosa* isolates without IMP genes were likely resistant due to either active drug efflux pump mechanisms (MexAB-OprM, MexEF-OprN, OprJ, OpmB, OpmH) or other classes of carbapenemase such as OXA-50.^6^^,^^7^

Colistin is the only effective antibiotic in particular cases for which *P. aeruginosa* is resistant to all tested antibiotics including the carbapenem group. The emergence of colistin-resistant strains posed a great threat to patients with severe infections.^30–32^ Our study found that eight (11%) of the *P. aeruginosa* isolates were resistant to colistin, compared with 7% (3%–13%) in *P. aeruginosa* isolates collected in 2012–13 in the Vietnam Resistance project (VINARES).^24^ Colistin resistance was found in all three hospitals, including three isolates of ST3151 from in Saint Paul in 2011 and 2012, two ST244 from Viet Duc and Saint Paul in 2014 and 2015, ST360 and ST654 from Thanh Nhan and Viet Duc hospitals in 2012–14 and ST773 from Saint Paul in 2012. All isolates were negative for *mcr* genes and are indicated in Figure 2 and Table S1. Resistant isolates that shared the same ST also shared very similar resistance gene profiles. This result places Vietnam in the group of countries with a high rate of colistin resistance. Our findings suggest cautious consideration of colistin use in treatment for carbapenem-resistant *P. aeruginosa* in clinical practice. Further studies on colistin resistance are needed.

The MLST data showed a high diversity of *P. aeruginosa* isolates. The 72 isolates were grouped into 18 different STs. Most of these STs were reported in previous studies: ST357 was primarily found in Asia and ST235 is recognized as a major international high-risk clone.^33–36^ Except for ST3151, all dominant STs (ST235, ST360, ST310, ST357) and four singletons (ST277, ST773, ST1420 and ST2166) have been reported before from Vietnam, carrying the same IMP genes.^16^ We also showed clustering of ST by hospital: particularly the fact that ST360 isolates were mainly found in Thanh Nhan Hospital and ST3151 was predominant in Saint Paul Hospital strongly suggested nosocomial transmission. Core genome phylogenetic and STs of the isolates were also in the same group. Some high-risk STs were found within a hospital (ST360) or in different hospitals (ST235, ST244, ST277, ST340, ST357) and in different years (Figures 2 and 3). We detected 14 isolates belonging to ST3151, an ST that we could only find one entry of in the MLST database and no mention of in the literature. ST3151 carried different carbapenem genes: *bla*_KPC-1_ (*n *=* *7), *bla*_IMP-15_ (*n *=* *1), and one isolate only carried *bla*_OXA-50_, suggesting that the *bla*_KPC-1_-positive *P. aeruginosa* strains in the study might have acquired *bla*_KPC-1_ from *K. pneumoniae* and *E. coli* through conjugative transfer as previously mentioned. These findings suggest that these STs may have been disseminated in healthcare settings in Vietnam and new drug-resistant STs could emerge under selective and antibiotic pressure.

A recent review shows that globally, from our frequently detected STs ST235, ST357 and ST773 continue to be described as international high-risk clones.^37^ Among IMP genes, *bla*_IMP-15_, _-26_ and _-51_ are still dominant in our study as previously described. These three have been previously described as important resistance genes in ST235 but not in ST357 or ST773, potentially reflecting local circulation and conjugation. KPC genes in *Pseudomonas* continue to be described, but rarely. We have no updated information on whether the KPC-producing ST3151 has been observed in Vietnam after January 2013.

Our study has some limitations that need to be addressed. Firstly, the sequencing from this work was conducted *post hoc* and the results from this isolate selection from 2011–15, given the diversity over space and time we see, will not be large and recent enough to represent current carbapenem-resistant *P. aeruginosa* in healthcare settings in Vietnam. Secondly, we were unable to assess the clinical significance of carbapenem-resistant *P. aeruginosa* regarding antibiotic treatment and outcome. Therefore, we propose that future studies should incorporate clinical data to obtain a better understanding of *P. aeruginosa* infections. Lastly, we only conducted genetic characterization of *P. aeruginosa* isolates and described the presence of genes but did not assess expression levels of resistance-associated genes, in which the case of efflux pumps and overexpression of AmpC may contribute to resistance.

Despite these limitations, our study confirms there is a high diversity with different levels of carbapenem resistance among clinical *P. aeruginosa* isolates. *bla*_IMP-26_ and other variants of IMP such as *bla*_IMP-15_ and *bla*_IMP-51_ are disseminated across healthcare settings in Vietnam. We found an association with several STs and these resistance genes and between combinations of genes and MICs. We also reported highly resistant ST1420 and ST3340 isolates co-harbouring *bla*_IMP-15_ and *bla*_DIM-1_, and seven ST3151 isolates carrying the *bla*_KPC_ gene, which could restrict available treatment options in healthcare settings. The 12 ST3151 isolates were detected in 2011–13, but not in 2014–15.

Our results provide a snapshot of the diversity of *P. aeruginosa* in time and space in hospitals in Hanoi, Vietnam, which is extremely important for a better understanding of the emergence and spread of drug resistance in Vietnam. Our sequencing data presented here was conducted *post hoc*, but the finding of a high diversity of resistant isolates in time and space makes it clear that real-time molecular surveillance of resistant isolates and their associated resistance mechanisms is necessary and useful to trace and help limit the spread of these isolates within and between hospitals and to inform infection prevention and control measures.

## Funding

This work was supported by a grant from Newton fund Vietnam (MRC: MR/N028317/1 and MOST: NHQT/SPDP/02.16); the Wellcome Trust of Great Britain, grant-in-aid from Japan Agency for Medical Research and Development (AMED), UK, Japan, and from Institut de Recherche pour le Développement (IRD) and Laboratoire Mixte International – “Drug Resistance in South East Asia” (LMI DRISA) for financial support.

## Transparency declarations

None to declare.

### Author contributions

Tran Huy Hoang, H. Rogier van Doorn and Tran Nhu Duong conceived the study and directed its implementation. Tran Huy Hoang, Keigo Shibayama, Masato Suzuki, Anne-Laure Bañuls, Vu Thi Ngoc Bich and Dang Duc Anh designed the study. Tran Nhu Duong, Pham Duy Thai, Luu Thi Vu Nga, Vu Phuong Thom, Trinh Hong Son and Tran Huy Hoang managed the implementation of the fieldwork, and Tran Huy Hoang, Tran Hai Anh, Tran Dieu Linh, Ngo Thi Hong Hanh, Nguyen Minh Thao, Trinh Khanh Linh, Vu Thi Ngoc Bich, Pham Ha My and Tran Van Anh undertook the laboratory work. Tran Huy Hoang, Trinh Son Tung, Le Viet Thanh, Vu Thi Ngoc Bich, Ngo Thi Hong Hanh, Tran Hai Anh and Lay-Myint Yoshida did analyses. Tran Huy Hoang, Tran Hai Anh, Vu Thi Ngoc Bich and H. Rogier van Doorn wrote the first draft of the paper. All the authors reviewed and edited drafts of the manuscript and approved the final version.

## Supplementary data

Table S1 and Figure S1 are available as Supplementary data at *JAC-AMR* Online.

## Supplementary Material

dlab103_Supplementary_Data
