# Supplementary material for: Resistance mechanisms and genetic relatedness among carbapenem-resistant Pseudomonas aeruginosa isolates from three major hospitals in Hanoi, Vietnam (2011–15)
Source: JAC Antimicrob Resist. 2021 Jul 27;3(3):dlab103. doi: 10.1093/jacamr/dlab103 (PMC8313516; doi:10.1093/jacamr/dlab103)
Supplement: dlab103_Supplementary_Data [file dlab103_supplementary_data.docx]

**Supplementary data**

**Table S1.** Resistant genotypes, phenotypes, sequence type of 72 *P. aeruginosa* isolates in three hospitals of Hanoi (*n*=72)

| **No** | ***WGS-ID*** | ***Hospital*** | ***ST*** | ***Phylogenic group*** | **Key antibiotic resistant genes** | | | | | | | | |  |  | **MIC (mg/L)** | | | | | | |
| --- | --- | --- | --- | --- | --- | --- | --- | --- | --- | --- | --- | --- | --- | --- | --- | --- | --- | --- | --- | --- | --- | --- |
|  |  |  |  |  | ***IMP-15*** | ***IMP-26*** | ***IMP-51*** | ***DIM-1*** | ***CARB-3*** | ***KPC-1*** | **OXA-50** | **QnrVC1** | ***Veb-1*** | ***PDC-*** | ***FosA*** | ***IMP*** | ***CIP*** | ***CAZ*** | ***AMK*** | ***GEN*** | ***AZT*** | ***CS*** |
| 1 | 41 | Viet Duc | 179 | IV | - | - | - | - | - | - | + | - | - | PDC-8 | + | R (16) | S (0.125) | S (4) | S (2) | S (1) | S (8) | S (0.5) |
| 2 | 61 | Viet Duc | 179 | IV | - | - | - | - | - | - | + | - | - | PDC-8 | + | R (128) | R (64) | R (64) | S (4) | S (2) | R (128) | S (0.25) |
| 3 | 13 | Viet Duc | 235 | IX | - | - | + | - | - | - | + | - | - | PDC-2 | + | R (32) | R (8) | R (256) | S (8) | R (32) | I (16) | S (0.25) |
| 4 | 17 | Viet Duc | 235 | IX | - | + | - | - | - | - | + | - | - | PDC-2 | + | R (128) | R (16) | R (>256) | R (64) | R (>128) | I (16) | S (0.5) |
| 5 | 42 | Thanh Nhan | 235 | IX | - | - | + | - | - | - | + | - | - | PDC-2 | + | R (32) | R (16) | R (256) | R (64) | R (64) | R (128) | S (0.5) |
| 6 | 43 | Thanh Nhan | 235 | IX | - | - | + | - | - | - | + | - | - | PDC-2 | + | R (32) | R (16) | R (256) | S (16) | R (64) | R (32) | S (1) |
| 7 | 46 | Viet Duc | 235 | IX | - | - | + | - | - | - | + | - | - | PDC-2 | + | R (32) | R (16) | R (256) | R (64) | R (64) | R (128) | S (1) |
| 8 | 47 | Viet Duc | 235 | IX | - | - | + | - | - | - | + | - | - | PDC-2 | + | R (32) | R (16) | R (256) | S (16) | R (64) | R (32) | S (2) |
| 9 | 59 | Saint Paul | 235 | IX | - | - | + | - | - | - | + | - | - | PDC-2 | + | R (16) | R (16) | R (256) | S (8) | R (64) | R (32) | S (0.5) |
| 10 | 60 | Viet Duc | 235 | IX | - | - | + | - | - | - | + | - | - | PDC-2 | + | R (16) | R (16) | R (256) | S (8) | R (64) | R (32) | S (0.25) |
| 11 | 69 | Viet Duc | 235 | IX | - | - | + | - | - | - | + | - | - | PDC-2 | + | R (16) | R (16) | R (128) | S (8) | R (32) | I (16) | S (0.5) |
| 12 | 75 | Viet Duc | 235 | IX | - | - | + | - | - | - | + | - | - | PDC-2 | + | R (16) | R(16) | R (256) | S (8) | R (32) | R (32) | S (0.5) |
| 13 | 77 | Viet Duc | 235 | IX | - | - | + | - | - | - | + | - | - | PDC-2 | + | R (64) | R (8) | R (256) | R (>256) | R  (>128) | R (32) | S (0.125) |
| 14 | 79 | Saint Paul | 235 | IX | - | - | + | - | - | - | + | - | - | PDC-2 | + | R (64) | R (8) | R (256) | R (>256) | R (>128) | R (32) | S (1) |
| 15 | 80 | Saint Paul | 235 | IX | - | - | + | - | - | - | + | - | - | PDC-2 | + | R (16) | R (16) | R (256) | S (8) | R (64) | R (32) | S (1) |
| 16 | 53 | Saint Paul | 244 | III | - | - | - | - | - | - | + | - | - |  | + | R (16) | S (0.25) | R (32) | S (4) | S (2) | I (16) | R (4) |
| 17 | 73 | Viet Duc | 244 | III | - | - | - | - | - | - | + | - | + | PDC-1 | + | R (8) | S (1) | R (>128) | R (>256) | R (32) | R (>128) | R (4) |
| 18 | 86 | Thanh Nhan | 244 | III | - | - | - | - | - | - | + | - | + | PDC-1 | + | R (16) | S (0.125) | R (>128) | S (2) | S (1) | S (8) | S (2) |
| 19 | 10 | Saint Paul | 245 | - | + | - | - | - | + | - | + | + | - | PDC-5 | + | R (32) | R (16) | R (128) | R (>256) | R (>128) | S (2) | S (1) |
| 20 | 85 | Thanh Nhan | 277 | II | - | - | - | - | - | - | + | - | - | PDC-5 | + | R (16) | S (0.125) | S (4) | S (2) | S (1) | S (8) | S (0.5) |
| 21 | 89 | Viet Duc | 277 | - | + | - | - | - | + | - | + | + | - | PDC-5 | + | R (32) | R (16) | R (64) | R (>256) | R (>128) | I (16) | S (1) |
| 22 | 99 | Thanh Nhan | 277 | II | - | - | - | - | - | - | + | - | - | PDC-3 | + | R (16) | S (0.125) | S (4) | S (2) | S (1) | S (8) | S (1) |
| 23 | 9 | Saint Paul | 310 | VIII | + | - | - | - | - | - | + | + | - | PDC-3 | + | R (32) | R (16) | R (128) | R (>256) | R (>128) | S (2) | S (2) |
| 24 | 19 | Saint Paul | 310 | VIII | - | - | - | - | - | - | + | - | - | PDC-3 | + | R (16) | S (0.125) | S (4) | S (2) | S (1) | S (8) | S (0.25) |
| 25 | 20 | Saint Paul | 310 | VIII | + | - | - | - | + | - | + | + | - | PDC-3 | + | R (64) | R (16) | R (128) | R (>256) | R (>128) | I (16) | S (0.5) |
| 26 | 28 | Viet Duc | 310 | VIII | + | - | - | - | + | - | + | - | - | PDC-7 | + | R (64) | R (8) | R (256) | R (>256) | R (>128) | S (8) | S (1) |
| 27 | 31 | Saint Paul | 310 | VIII | - | - | - | - | + | - | + | - | - | PDC-3 | + | R (16) | S (0.25) | R (32) | S (4) | S (2) | I (16) | S(0.5) |
| 28 | 88 | Thanh Nhan | 310 | VIII | - | - | - | - | - | - | + | - | - | PDC-3 | + | R (16) | S (0.125) | S (4) | S (2) | S (1) | S (8) | S (0.125) |
| 29 | 7 | Saint Paul | 310 | VIII | + | - | - | - | + | - | + | + | - | PDC-3 | + | R (32) | R (16) | R (128) | R (>256) | R (>128) | S (2) | S (0.5) |
| 30 | 68 | Viet Duc | 313 | - | - | - | - | - | - | - | + | - | - | PDC-7 | + | R (16) | S (0.25) | R (32) | S (4) | S (2) | I (16) | S (0.5) |
| 31 | 22 | Saint Paul | 357 | VI | + | - | - | - | + | - | + | + | - | PDC-3 | + | R (64) | R (4) | R (64) | R (>256) | R (>128) | S (8) | S (0.125) |
| 32 | 34 | Viet Duc | 357 | VI | + | - | - | - | + | - | + | + | - | PDC-3 | + | R (128) | R (16) | R (256) | R (>256) | R (>128) | I (16) | S (0.5) |
| 33 | 35 | Saint Paul | 357 | VI | + | - | - | - | + | - | + | + | - | PDC-3 | + | R (64) | R (16) | R (128) | R (>256) | R (>128) | I (16) | S (1) |
| 34 | 37 | Viet Duc | 357 | VI | + | - | - | - | + | - | + | + | - | PDC-3 | + | R (64) | R (8) | R (256) | R (>256) | R (>128) | R (32) | S (2) |
| 35 | 78 | Saint Paul | 357 | VI | + | - | - | - | + | - | + | + | - | PDC-3 | + | R (64) | R (16) | R (128) | R (>256) | R (>128) | I (16) | S (2) |
| 36 | 84 | Viet Duc | 357 | VI | + | - | - | - | + | - | + | + | - | PDC-3 | + | R (64) | R (128) | R (256) | S (8) | R (>128) | I (16) | S (2) |
| 37 | 97 | Thanh Nhan | 357 | VI | - | - | - | - | + | - | + | - | - | PDC-3 | + | R (16) | S (0.125) | S (4) | S (2) | S (1) | S (8) | S (0.5) |
| 38 | 8 | Thanh Nhan | 360 | I | + | - | - | - | + | - | + | + | - | PDC-5 | + | R (32) | R (16) | R (128) | R (>256) | R (>128) | S (2) | S (1) |
| 39 | 18 | Thanh Nhan | 360 | I | + | - | - | - | + | - | + | + | - | PDC-5 | + | R (64) | R (16) | R (128) | R (>256) | R (>128) | I (16) | S (0.5) |
| 40 | 21 | Thanh Nhan | 360 | I | + | - | - | - | + | - | + | + | - | PDC-5 | + | R (64) | R (16) | R (128) | R (>256) | R (>128) | I (16) | S (0.25) |
| 41 | 23 | Thanh Nhan | 360 | I | + | - | - | - | + | - | + | + | - | PDC-5 | + | R (64) | R (16) | R (128) | R (>256) | R (>128) | I (16) | S (0.25) |
| 42 | 76 | Thanh Nhan | 360 | I | + | - | - | - | + | - | + | + | - | PDC-5 | + | R (64) | R (16) | R (128) | R (>256) | R (>128) | I (16) | R (>16) |
| 43 | 81 | Thanh Nhan | 360 | I | - | - | - | - | - | - | + | - | - | PDC-5 | + | R (16) | S (0.125) | S (4) | S (2) | S (1) | S (8) | S (0.5) |
| 44 | 87 | Thanh Nhan | 360 | I | + | - | - | - | + | - | + | + | - | PDC-5 | + | R (32) | R (16) | R (128) | R (>256) | R (>128) | S (2) | S (2) |
| 45 | 91 | Thanh Nhan | 360 | I | + | - | - | - | + | - | + | + | - | PDC-5 | + | R (32) | R (16) | R (128) | R (>256) | R (>128) | S (2) | S (0.5) |
| 46 | 92 | Thanh Nhan | 360 | I | - | - | - | - | + | - | + | - | - | PDC-5 | + | R (16) | S (0.125 | S (4) | S (2) | S (1) | S (8) | S (0.5) |
| 47 | 93 | Thanh Nhan | 360 | I | + | - | - | - | + | - | + | + | - | PDC-5 | + | R (32) | R (16) | R (128) | R (>256) | R (>128) | S (2) | S (1) |
| 48 | 38 | Viet Duc | 654 | - | - | + | - | - | - | - | + | - | - | PDC-3 | + | R (>128) | R (32) | R (>256) | R (>256) | R (>128) | R (32) | R (4) |
| 49 | 27 | Saint Paul | 773 | VIII | + | - | - | - | + | - | + | + | - | PDC-2 | + | R (64) | R (16) | R (128) | R (>256) | R (>128) | I (16) | S (0.25) |
| 50 | 29 | Saint Paul | 773 | VIII | + | - | - | - | + | - | + | + | - | PDC-2 | + | R (32) | R (16) | R (128) | R (>256) | R (>128) | S (2) | S (1) |
| 51 | 33 | Viet Duc | 773 | VIII | + | - | - | - | + | - | + | + | - | PDC-2 | + | R (64) | R (16) | R (128) | R (>256) | R (>128) | S (4) | S (0.125) |
| 52 | 95 | Saint Paul | 773 | VIII | + | - | - | - | + | - | + | + | - | PDC-2 | + | R (64) | R (16) | R (256) | R (>256) | R (>128) | S (4) | R (8) |
| 53 | 56 | Saint Paul | 856 | - | - | - | - | - | - | - | + | - | + | PDC-5 | + | R (16) | S (0.125) | R (>128) | S (2) | S (1) | S (8) | S (1) |
| 54 | 36 | Saint Paul | 1420 | - | + | - | - | + | + | - | + | - | - | PDC-7 | + | R (>128) | R (16) | R (>256) | S (16) | R (>128) | R (128) | S (1) |
| 55 | 14 | Viet Duc | 2166 | - | + | - | - | - | + | - | + | + | - | PDC-5 | + | R (64) | R (4) | R (64) | R (>256) | R (>128) | S (8) | S (2) |
| 56 | 2 | Saint Paul | 3151 | V | - | - | - | - | + | + | + | + | - | PDC-7 | + | R (>128) | R (8) | R (32) | R (>256) | R (>128) | R (>128) | S (1) |
| 57 | 3 | Saint Paul | 3151 | V | - | - | - | - | + | + | + | + | - | PDC-7 | + | R (>128) | R (8) | R (32) | R (>256) | R (>128) | R (>128) | S (2) |
| 58 | 4 | Saint Paul | 3151 | V | - | - | - | - | + | + | + | + | - | PDC-7 | + | R (>128) | R (8) | R (32) | R (>256) | R (>128) | R (>128) | R (4) |
| 59 | 5 | Viet Duc | 3151 | V | + | - | - | - | + | - | + | + | - | PDC-7 | + | R (32) | R (16) | R (128) | R (>256) | R (>128) | S (2) | S (0.125) |
| 60 | 6 | Saint Paul | 3151 | V | + | - | - | - | + | - | + | + | - | PDC-7 | + | R (32) | R (16) | R (128) | R (>256) | R (>128) | S (2) | R (4) |
| 61 | 11 | Saint Paul | 3151 | V | - | - | - | - | + | + | + | + | - | PDC-7 | + | R (>128) | R (8) | R (32) | R (>256) | R (>128) | R (>128) | S (0.5) |
| 62 | 15 | Viet Duc | 3151 | V | + | - | - | - | + | - | + | + | - | PDC-7 | + | R (32) | R (16) | R (128) | R (>256) | R (>128) | S (2) | S (0.125) |
| 63 | 26 | Saint Paul | 3151 | V | + | - | - | - | + | - | + | + | - | PDC-7 | + | R (32) | R (16) | R (128) | R (>256) | R (>128) | S (2) | S (0.5) |
| 64 | 32 | Saint Paul | 3151 | V | + | - | - | - | + | - | + | + | - | PDC-7 | + | R (64) | R (16) | R (128) | R (>256) | R (>128) | I (16) | R (8) |
| 65 | 39 | Saint Paul | 3151 | V | + | - | - | - | + | - | + | + | - | PDC-7 | + | R (64) | R (16) | R (128) | R (>256) | R (>128) | I (16) | S (1) |
| 66 | 57 | Saint Paul | 3151 | V | - | - | - | - | + | + | + | - | - | PDC-7 | + | R (>128) | R (8) | R (32) | R (>256) | R (>128) | R (>128) | S(0.125) |
| 67 | 62 | Saint Paul | 3151 | V | - | - | - | - | + | + | + | + | - | PDC-7 | + | R (>128) | R (8) | R (32) | R (>256) | R (>128) | R (>128) | S (0.125) |
| 68 | 71 | Saint Paul | 3151 | V | - | - | - | - | + | + | + | - | - | PDC-7 | + | R (>128) | R (8) | R (32) | R (>256) | R (>128) | R (>128) | S (0.5) |
| 69 | 72 | Saint Paul | 3151 | V | - | - | - | - | + | - | + | - | - | PDC-7 | + | R (16) | S (0.125) | S (4) | S (2) | S (1) | S (8) | S (1) |
| 70 | 90 | Thanh Nhan | 3308 | - | + | - | - | - | + | - | + | + | - | PDC-7 | + | R (32) | R (16) | R (128) | R (>256) | R (>128) | S (2) | S (0.125) |
| 71 | 82 | Viet Duc | 3440 | - | + | - | - | + | - | - | + | + | - | PDC-3 | + | R (>128) | R (16) | R (>256) | S (16) | R (>128) | R (128) | S (0.125) |
| 72 | 96 | Thanh Nhan | 3361 | - | - | - | - | - | - | - | + | - | - | PDC-3 | + | R (16) | S (0.125) | S (4) | S (2) | S (1) | S (8) | S(1) |

Note: S: Sensitive; I: Intermidiate; R: Resistan; ST; Sequence type

**Figure S1.** Distribution of IMP gene by Hospital 2011-2015. Saint Paul (A); Thanh Nhan (B); Viet Duc (C).
